# Editorial

**Published:** 1838-09-26

**Authors:** 


### THE MEDICAL EXAMINER.

PHILADELPHIA, SEPT. 26, 1838.

Dr. W. W. Gerhard has been associated with the present Editors, in the conduct of this Journal. This connection has been formed with particular reference to a contemplated change in the periodical character of the Journal. It is proposed to publish it, from the commencement of the ensuing year, weekly instead of fortnightly. This was the original intention of the Editors, in the event of the success of the undertaking, and, in now determining to act upon it, they comply with the expressed wishes of a large proportion of their subscribers. The practical character of the Journal, which has mainly contributed to render it acceptable, it will be a principal aim of the Editors to preserve. Reports of clinical and of other lectures, and of hospital cases, will, as heretofore, occupy a large space in the columns of the Examiner. To other departments, existing arrangements will, it is believed, give increased efficiency. An European correspondence has been established, by which our readers will be presented with a regular series of communications, from a physician of extensive connections with the public institutions of Paris. We hope to be able to commence the publication of these letters, during the current year, and venture to assure our readers, in anticipation, that they will find them to contain a faithful and interesting picture of the actual condition of the medical science, in the French metropolis, and throughout the Continent of Europe. We need hardly add, that from our ample original American sources we shall continue to draw as largely as heretofore, and we take the occasion again to solicit the contributions of the numerous and respectable body of physicians throughout the country, among whom this Journal is circulated.

*Extracts of a letter from Dr.* Rufz, *of the Island of Martinique.*

"It would perhaps be interesting for you to know, that, from the year 1826, the yellow fever has ceased to appear as an epidemic. From time to time, we have a few sporadic cases amongst the soldiers of the garrison. The only circumstance, which can account for this cessation, is the fact, that our intercourse with Europe has greatly diminished, and, of course, the number of unacclimated strangers is much less considerable. * * * * In my private practice, I have seen no case of typhoid fever,—but I recollect to have seen it twice in soldiers at the military hospital. We have dysenteries, and a great number of diarrhœas, which are generally regarded as consequences of dysentery,—as to these tropical affections, there are still many points wanting to complete their history.

"The other diseases that we see here differ but little from those of France, and I believe that the physician may almost add with the poet—

'Nec animum, nec corpus, mutant, qui trans mare currunt.'"

Dr. Rufz is an agrégé of the Faculty of Paris, and one of those best fitted to observe disease, in the most conscientious manner. We regret that he did not render his letter still more valuable, by giving us the results of his observation in remittent and intermittent fevers, which, with dysentery and diarrhœa, constitute the chief endemic affections of the West Indies, as well as of our Southern states.

We regard our foreign and domestic correspondence as one of the most valuable departments of our journal, and are fully convinced that it will materially increase our knowledge of the diseases endemic in various sections of the United States. We are particularly desirous to obtain complete accounts of the diseases, peculiar to the Southern as well as to the Western states, and will, at all times, be obliged to our correspondents for such information as they may possess, respecting the peculiar features and best modes of treating the diseases, which are endemic in the part of the country in which they reside. The trouble which these contributions would require is but of little moment, when compared with the instruction that each practitioner would receive from reviewing the cases offered to his observation, and with the inestimable benefits which such a course would confer upon the profession. The frequent publication of our journal, with its eminently practical character, affords great facilities for the rapid diffusion of knowledge of this kind, and we sincerely hope that our medical friends will not disappoint us in the expectations in which we reasonably indulge, of receiving many valuable contributions from practitioners, who, under ordinary circumstances, would have preserved no record of the facts which they constantly meet with. The benefits which this record of facts will confer, must rapidly increase, and will keep pace with their accumulation. In proportion as they become more numerous, every one who is interested in science will be enabled to render his future observations more useful, from comparing them with those of analagous kinds which have presented themselves to his professional brethren; while the number of points to be ascertained by future observation will be known in a more definite manner, and the inquiries of a physician may be directed with more precision and with less trouble to himself. We shall not be wanting in our efforts to aid in these researches, by a careful comparison of the documents which we may from time to time receive, and will present them to our readers in a sort of "digest," as often as they may be accumulated in sufficient numbers.

One more remark we must make, before concluding our request for the aid of our colleagues, in undertaking so important a task as that of collecting a complete account of the diseases most frequent in each section of the country. It is necessary that their letters should contain sufficient fulness of detail to convince us that our correspondents have observed with the sort of care, and the degree of previous knowledge, necessary to render their observations useful;—of their perfect good faith, we would, of course, entertain no doubts. But, on the other hand, it is equally necessary that we should extract just so much from each communication as may be of important use to the reader,—in other words, we will be obliged to condense our materials into the most concise form. If our correspondents should possess sufficient leisure to perform this task for us, we shall be doubly indebted to them. Many of our medical friends have already promised communications of such points as will most interest them. We would remind them of their intentions, at the same time that we earnestly request the co-operation of the profession generally, in a task, which, if properly supported, cannot fail to be of immense benefit.

The remarks which we have just made, particularly refer to those shorter communications which may be comprised within the limits of a letter; we would not, however, be understood to mean, that more elaborate communications, if not too long for the limits of the Examiner, would be less desirable. These will doubtless be furnished, from time to time, and will always be published in as short a period as possible from the date of their reception.

